# Psychometric Properties of the Turkish Version of the Dietary Fat and Free Sugar-Short Questionnaire (DFS-TR) in Adults: A Validity and Reliability Study

**DOI:** 10.3390/nu18030421

**Published:** 2026-01-27

**Authors:** Çiler Özenir, Mihrican Çubuk, Canan Altınsoy, Duygu Ağagündüz

**Affiliations:** 1 Department of Nutrition and Dietetics, Kırıkkale University, 71100 Kırıkkale, Türkiye; cileraslanalp@kku.edu.tr; 2 Department of Nutrition and Dietetics, Erzincan Binali Yıldırım University, 24100 Erzincan, Türkiye; mihrican.kacar@erzincan.edu.tr; 3 Department of Nutrition and Dietetics, Recep Tayyip Erdoğan University, 53350 Rize, Türkiye; 4 Department of Nutrition and Dietetics, Gazi University, 06490 Ankara, Türkiye; duyguturkozu@gazi.edu.tr

**Keywords:** dietary assessment, dietary intake, dietary fat, dietary free sugar, reliability, saturated fat, validity

## Abstract

**Background/Objectives:** The aim of this study was to translate the Dietary Fat and Free Sugar-Short Questionnaire (DFS) into Turkish (DFS-TR) and to establish its construct validity and reliability. **Methods:** Quota sampling was used to ensure demographic homogeneity across gender and age groups. Participant distribution by age categories was proportionally aligned with the demographic statistics of the adult Turkish population. The study comprised 314 participants aged 19–64 years (38.78 ± 12.10), of which 54.5% were female. The data collection form consisted of demographic characteristics, anthropometric measurements, information on eating habits, DFS-TR, the Food Frequency Questionnaire (FFQ), and the Power of Food Scale (PFS). **Results:** Test–retest reliability was confirmed in the 314 participants with a 4-week interval (r = 0.997, *p* < 0.01). The Cronbach α reliability coefficient was α = 0.777. Using the split-half method, the correlation coefficient between the two halves was 0.681, the Spearman–Brown coefficient was 0.811, and the Guttman coefficient was 0.809. Participants’ total DFS-TR scores and sub-dimension scores varied according to age, education level, income level, anthropometric characteristics, physical activity, and dietary habits, but did not vary according to gender or marital status. To investigate convergent validity, participants’ DFS-TR scores were correlated with the FFQ and PFS. DFS-TR scores showed a significant correlation with the percentage of energy from saturated fat and free sugar assessed by the FFQ. Positive relationships were found between DFS-TR scores and the total PFS score, particularly with the sub-dimension scores for food available and food present. **Conclusions:** The DFS-TR can be used as a reliable and valid measurement tool for estimating saturated fat and free sugar intake among Turkish adults.

## 1. Introduction

Non-communicable diseases (NCDs), such as heart disease, stroke, cancer, diabetes, and chronic lung conditions, represent a significant and increasing global health challenge, accounting for 74% of all deaths worldwide. The burden of NCDs is disproportionately concentrated in low-income and middle-income nations, where more than 86% of the 17 million premature deaths—those occurring before age 70 years—take place [1,2]. Excess weight and obesity, high blood lipid and glucose concentrations, and hypertension are considered the four main modifiable risk factors for NCDs, and most of these are closely linked to dietary habits. Therefore, improving dietary patterns has become a central focus of current prevention and treatment strategies aimed at reducing excess body weight and NCD-related morbidity. Two potential targets for dietary changes are saturated fat and free sugars [3,4]. Accordingly, reducing saturated fat and free sugar intake remains a priority goal in international dietary guidelines, as emphasized by World Health Organization (WHO) recommendations and global NCD action plans [5,6].

Sugar has forever been part of the human diet. The consumption of free sugars—one of several types of sugars—has been linked to a higher risk of chronic diseases, such as diabetes, obesity, and cardiovascular disease [7]. To reduce the risks of various chronic conditions, especially obesity and dental caries, WHO issued a recommendation in 2015 to limit free sugar intake. Free sugar is defined as sugar added to foods during production or cooking and sugars found in honey, syrups, and fruit juices. The guideline recommends reducing free sugar intake to less than 10% of daily energy intake and less than 5% for additional health benefits [8,9]. Consuming free sugars has been shown to increase a food’s energy density without enhancing satiety or providing nutrients, and sugars (e.g., fructose) can lead to decreased circulating insulin and leptin levels, along with increased postprandial ghrelin concentrations [10]. It has also been suggested that sugar can cause weight gain, higher serum lipids, and fatty internal organs [7,11]. Another factor, dietary fat, is the macronutrient with the highest energy per gram, and high intake of saturated fat is believed to elevate the risk of cardiovascular disease and contribute to obesity by raising low-density lipoprotein levels [12]. As a result, eating foods and beverages that are high in free sugars and saturated fats is associated with poor health outcomes. Accurately measuring free sugar and saturated fat intake can help understanding their health effects and help develop more effective public health policies [13].

Saturated fats and added sugars contribute considerably to total energy intake in many populations. Common food sources include cheese, pizza, ice cream, soft drinks, and sweet baked goods [14]. Accurate identification of foods high in fat and added sugars is essential in dietary behavior research because these foods are key contributors to poor diet quality and adverse health outcomes, including metabolic and cognitive dysfunction [15].

Commonly used methods for assessing dietary habits and food intake include diet diaries, 24 h recall dietary records, and the Food Frequency Questionnaire (FFQ). These methods may provide reliable estimates of daily food intake [4]. However, traditional methods each present inherent constraints and might not always capture nuanced variations in high-fat and high-sugar food consumption due to recall bias and reporting limitations. FFQs are widely used due to their practicality in large-sample studies, but evidence suggests they might underestimate or misclassify actual fat and nutrient intakes compared with more controlled dietary records [16]. However, FFQs might not be appropriate for large-scale epidemiological studies and rapid assessments. In this context, new methods are being developed to address this need [17]. It is essential to use appropriate and valid assessment tools, such as the Dietary Fat and Free Sugar Short Questionnaire (DFS), to estimate the proportion of free sugars and saturated fat in a diet [4,17]. The Dietary Fat and Sugar (DFS) questionnaire is a brief self-report screening tool developed by Francis and Stevenson in 2013 to assess habitual consumption of foods rich in saturated fat and free sugars over the previous 12 months. Originally developed in English, the DFS was first validated in a university-based sample at Macquarie University, Sydney, Australia [17]. The questionnaire consists of 26 items structured as a checklist. The first 25 items assess the frequency of consumption of foods from different food groups, while the final item evaluates the amount of sugar added to beverages or foods. The DFS comprises three sub-dimensions: foods contributing predominantly saturated fat, foods contributing predominantly free sugars, and foods contributing both saturated fat and free sugars, allowing for the independent and combined evaluation of these dietary components. The DFS has demonstrated acceptable internal consistency and convergent validity with dietary records and food frequency questionnaires. Moreover, scores exceeding established cut-off values have been shown to reflect intakes above WHO recommendations for saturated fat and free sugars [17]. Owing to its concise structure and ease of administration, the DFS enables the rapid identification of individuals with high dietary exposure to these nutrients, which are closely associated with cardiometabolic and inflammatory risk [4,17,18].

In the recent literature, the DFS has been utilized as a practical and time-efficient alternative to more comprehensive dietary assessment methods, demonstrating its applicability in studies examining associations between habitual fat and sugar intake, mental health outcomes, and cognitive performance [19,20]. To date, apart from the original English version, only German and Polish cultural adaptations of the DFS have been formally validated and published [4,17,18], and no validated Turkish version is currently available.

Therefore, the present study aimed to evaluate the validity and reliability of the Turkish version of the Dietary Fat and Sugar questionnaire (DFS-TR). The psychometric properties of the DFS-TR and its relative validity in comparison with a comprehensive food frequency questionnaire were examined.

## 2. Materials and Methods

This cross-sectional study included Turkish adults aged 19–64 years who met the inclusion criteria and volunteered to participate in Ankara. Based on available information, the sample size should be five to ten times the number of items in the questionnaire, according to validity and reliability guidelines [21]. For this questionnaire with 26 items, the calculated sample size was 312, assuming ten times the number of items, a possible 260 participants, and 20% attrition. However, there are differing opinions in the literature about how large a sample should be for developing measurement tools and assessing validity and reliability. Kass and Tinsley (1979) [22] recommend at least 300 participants or five to ten times the number of items, while Comrey and Lee (1992) [23] suggest 300–500 for reliable factor analysis results. Based on the questionnaire items, the target of at least 300 participants was met, with a total of 314 participants. The study used quota sampling to ensure demographic consistency across gender and age groups. Participants were distributed across age groups (20–24, 25–29, 30–34, 35–39, 40–44, 45–49, 50–54, 55–59, and 60–64 years) following population data from the Turkish Statistical Institute [24], and the number in the sample was reached at a rate appropriate to the age group.

*Inclusion Criteria:*

To be included in the study, participants had to be aged 19–64 years, be literate, and not meet any of the exclusion criteria.

*Exclusion Criteria:*

Participants were excluded if they were younger than 19 years or 65 years or older, had metabolic or systemic diseases that affect nutrient intake (e.g., diabetes and thyroid disorders), had a history (past or current) of neurological or psychiatric diagnoses, had a daily energy intake in excess of 4000 kcal or less than 800 kcal, used simple sugar or fat-based dietary supplements, had a history of neurological or psychiatric diagnosis (past or current), had a fat or sugar-restricted diet within the last year, were pregnant or undergoing lactation, were elite athlete status, had a physical or cognitive disability, had an eating disorder diagnosis, consumed more than 20 g of alcohol per day, had a history of substance abuse, or were a student or specialist in nutrition or dietetics.

*Data Collection Tools:*

Data for the study were collected via a face-to-face questionnaire that was conducted directly by the researchers.

*Personal Information Form:*

Participants’ sociodemographic characteristics, health history, and dietary habits, among other factors, were gathered via the questionnaire. This questionnaire included questions regarding date of birth, gender, marital status, education level, occupation, income level, health status, physical activity level, anthropometric measurements (self-reported body weight and height), and dietary habits (main and snack meal consumption, eating out, snack preferences, etc.).

*Dietary Fat and Free Sugar-Short Questionnaire (DFS):*

This 26-item self-reporting tool was developed by Francis and Stevenson [17] in 2013 to assess the frequency of dietary intake of foods high in saturated fat and free sugars spanning the past year. The first 25 items relate to the consumption of food from different food groups. The last item concerns adding sugar to foods or drinks consumed daily. Responses were scored on a questionnaire ranging from 1 (less than once a month) to 5 (5 times a week or more). For the last item on the amount of sugar added to food and beverages, the scoring ranged from 1 (none) to 5 (7 times or more). The questionnaire consisted of three sub-dimensions: saturated fat intake only (items 1–12), free sugar intake only (items 17, 18, 20, 21, 23, 24, and 26), and saturated fat plus free sugar intake (items 13–16, 19, 22, and 25). In the original study, the scoring of the options was graded from 1 to 5, with a minimum total score of 26 and a maximum total score of 130. Individuals with questionnaire scores >60 had mean estimates for saturated fat and sugar intake higher than WHO recommendations (total energy from fat <30%, saturated fat <10%, and free sugars <10%). Therefore, the questionnaire has the potential to be used as a screening tool, and scores greater than 60 can be interpreted as identifying individuals who should be advised to reduce their intake of these nutrients. In the original study, the Cronbach’s α of 0.76 was considered acceptable [17].

*Food Frequency Questionnaire (FFQ):*

Dietary intake data were collected using an FFQ to assess participants’ consumption patterns of 89 food items spanning the past year. The questionnaire evaluates how often foods were eaten with predefined categories: never, once a month, once every 15 days, 1–2 times per week, 3–4 times per week, 5–6 times per week, daily, or at every meal. For each chosen food intake frequency, participants reported their typical portion size per eating occasion. Daily intake amounts were calculated by multiplying the reported portion size (in standardized household measures or g/mL) by frequency-specific coefficients. Portion-size estimation was supported with the Food and Nutrient Photo Catalog [25]. Data were analyzed with the BEBIS (version 9) Nutrition Information System software to calculate nutrient and energy intake values.

*Power of Food Scale (PFS):*

In this study, the aim was to examine associations between nutrition and health status and PFS and DFS. The PFS was developed by Lowe et al. in 2009, and the validity and reliability of the Turkish version were examined by Ülker et al. in 2020 [26,27]. The Turkish version of the scale consists of 13 questions and has three sub-dimensions: food available, food present, and food taste. The first sub-dimension (items 1, 2, 9, and 10) is for “food available”, which evaluates general thoughts about food. The second sub-dimension (items 3–6) is for “food present”, which assessed attraction to the food the individual has direct access to. The third sub-dimension (items 7, 8, and 11–13) is for “food taste”, which measures the desire and pleasure obtained from tasting food for the first time. The assessment was based on a 5-point Likert scale (1 = strongly disagree, 2 = disagree, 3 = undecided, 4 = agree, and 5 = strongly agree). At the end of the evaluation, four scores were obtained, including three sub-dimension scores and a total scale score. Total and sub-dimension scores were calculated by summing the item scores and dividing by the number of items. For example, the total score was obtained by dividing the sum of all 13 items by 13, and the food availability score was calculated by dividing the scores from items 3, 4, 5, and 6 by 4. The higher the score on the scale, the greater the predisposition to hedonic hunger. Cronbach’s α coefficient for PFS-TR was 0.922, and Cronbach’s α for food available, food present, and food taste were 0.849, 0.797, and 0.82, respectively [27].

*Anthropometric Measurements:*

Body weight (kg) and height (cm) were obtained from self-report. Body mass index (BMI) was calculated using the formula body weight/(height)^2^ (kg/m^2^) and categorized according to the WHO classification for adults. Participants were classified as follows: underweight (BMI < 18.50 kg/m^2^), normal weight (18.50–24.99 kg/m^2^), overweight (25.00–29.99 kg/m^2^), or obese (≥30.00 kg/m^2^) [28].

*Translation Phase:*

The English-language DFS was translated into Turkish via a multi-stage process. First, four independent bilingual experts in nutrition and dietetics performed forward translations. The translated items were then evaluated by four independent linguists and 20 experts in nutrition and dietetics, who rated the semantic and contextual appropriateness of each item using a 5-point questionnaire. Items scoring ≤3 were revised collaboratively by the translators and linguists. Subsequently, Turkish language experts assessed the linguistic clarity and cultural relevance of the translated version. Items in the original questionnaire that included food not commonly present in Turkish culture, particularly pork, were revised in a culturally appropriate manner without reducing the number of items.

For back-translation, three independent bilingual translators (blinded to the original English version) converted the Turkish questionnaire back into English. The back-translated version was systematically compared with the original English text to identify discrepancies in meaning. Items with non-trivial semantic deviations were flagged and re-evaluated by a panel of nutrition and dietetics experts fluent in both languages. Minor adjustments were made to resolve ambiguities and ensure conceptual equivalence. Lastly, the linguistic and content validity of the Turkish version was formally assessed using Davis’s technique [29].

*Pilot Test:*

A pilot study was conducted using the translated questionnaire, with a planned sample of 60 participants. For this preliminary analysis, internal consistency (Cronbach’s α) and item-total correlation coefficients were calculated. The internal consistency coefficient was evaluated against a threshold of ≥0.70 and item-total correlations were examined to identify values less than 0.30. Items with correlations ≥0.20 were retained but flagged for review, while items with correlations <0.20 were considered for removal to improve questionnaire performance. During the pilot test, participants did not report major difficulties in understanding the items; without removing any items, minor explanations were added in parentheses to improve clarity. After these adjustments, the finalized questionnaire was subjected to further validity and reliability analyses.

*Data Analysis:*

Data analyses were conducted in accordance with the predefined aims of the study, following a structured validation framework. Descriptive statistics were first calculated to summarize participants’ sociodemographic characteristics, lifestyle-related variables, and DFS-TR scores.

The reliability of the DFS-TR was assessed by examining internal consistency and temporal stability. Internal consistency was evaluated using Cronbach’s α coefficients for the total scale and sub-dimensions. Split-half reliability was assessed using Spearman–Brown and Guttman coefficients. Test–retest reliability was examined by re-administering the DFS-TR to the same participants after a four-week interval and calculating correlation coefficients between the two administrations.

Criterion-related validity was evaluated by assessing the association between DFS-TR scores and dietary intake data obtained from the FFQ, including total energy intake and the percentage of energy derived from total fat, saturated fat, sucrose, and free sugars. Construct validity was further examined using known-groups comparisons by analyzing differences in dietary intake across quartiles of DFS-TR scores. Convergent validity was assessed by examining correlations between DFS-TR scores and the PFS, which measures hedonic hunger.

Data distribution was assessed using skewness and kurtosis values. Depending on the distribution of the data, parametric or non-parametric statistical tests were applied. Categorical variables were expressed as number (n) and percentage (%), while continuous variables were presented as mean ± standard deviation or median (minimum–maximum). All statistical analyses were performed using SPSS (Statistical Package for Social Sciences) version 23.0, and a *p*-value < 0.05 was considered statistically significant.

*Ethical Consideration:*

Permission was obtained from Gazi University Ethics Commission dated 30 July 2024 and numbered 2024-1204. Written informed consent was obtained from the participants who volunteered for the study in accordance with the Declaration of Helsinki.

## 3. Results

### 3.1. Participant Characteristics

The distribution of demographic characteristics of the participants is provided in Table 1. The average age of the participants was 38.78 ± 12.10 years, 54.5% of whom were female. When examining educational status, 38.5% were university graduates. A total of 63.4% of participants were married, and 66.2% had an income equal to their expenses. Only 18.8% of the participants engage in regular physical activity. Among those who do physical activity, the most preferred type was walking at 87.3%. Regarding BMI distribution, 55.7% of the participants were in the normal weight range, 29.6% were overweight, 10.5% were obese, and 4.2% were underweight.

### 3.2. Participants’ DFS-TR Scores According to Some Parameters

Participants’ total DFS-TR scores and scores on the fat and sugar sub-dimensions varied by age (*p* < 0.05). The 25–29 age group had lower total scores than the 40–44, 45–49, and 50–54 age groups; scored lower than the 35–39, 40–44, 45–49, and 60–64 age groups for the fat sub-dimension; and scored significantly lower than the 45–49, 50–54, and 60–64 age groups for the sugar sub-dimension. There was no significant difference between age groups in the fat + sugar sub-dimension (*p* > 0.05). In addition, there was no difference in participants’ total DFS-TR scores and sub-dimension scores based on gender or marital status (*p* > 0.05). There were significant differences in total scores and fat and sugar sub-dimensions between groups based on participants’ education level (*p* < 0.05). Individuals with an income that equaled their expenses scored higher than those with an income that exceeded their expenses in both sugar and fat + sugar sub-dimensions (*p* < 0.05). Those who engage in regular physical activity had a significantly lower total score than those who do not engage in physical activity and a lower sugar sub-dimension score than both those who do not engage and those who occasionally engage in physical activity (*p* < 0.05). Participants with class 1 obesity had higher total DFS-TR and sugar sub-dimension scores compared with individuals with normal weight and overweight. Also, those with class 1 obesity scored higher than those with normal weight on the fat and fat + sugar sub-dimension (*p* < 0.05; Table 2).

Individuals who ate meals outside the home sometimes scored higher for total score, fat sub-dimension, and fat plus sugar sub-dimension than those who occasionally ate meals outside the home (*p* < 0.05). Participants who frequently used frying as a cooking method had higher total DFS-TR scores, sugar sub-dimension scores, and fat + sugar sub-dimension scores than those who preferred boiling, baking, grilling, or roasting. Those who frequently preferred the frying method scored higher on the fat sub-dimension than those who preferred the grilling method (*p* < 0.05; Table 2).

**Figure 1:**
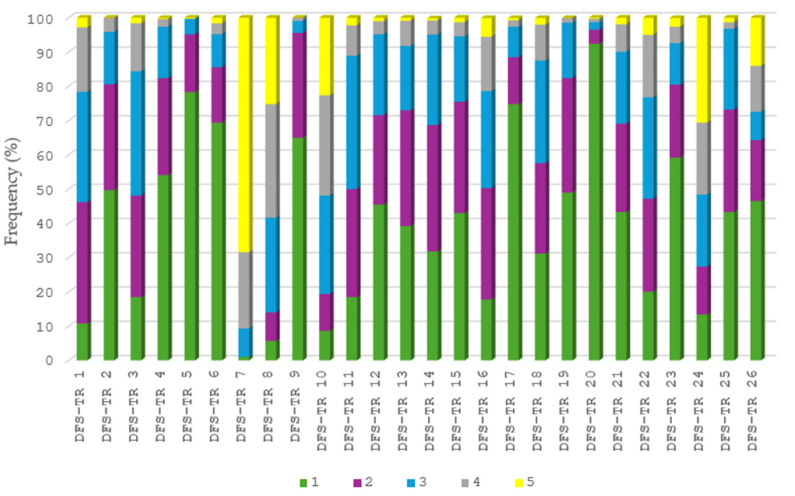
Item-Based Frequency Patterns of the DFS-TR in Adults.

The frequency distributions of participants’ responses to each of the 26 items of the DFS-TR are presented in Figure 1. For further information and evaluations for Figure 1, items of the DFS-TR are provided in Supplementary Tables S1 and S2.

In the figure, response option 1 represents ‘once a month or less’ for items 1–25, whereas it represents ‘none’ for item 26. Option 2 corresponds to ‘2–3 per month’ for items 1–25 and to ‘1–2’ for item 26. Option 3 corresponds to ‘1–2 per week’ for items 1–25 and to ‘3–4’ for item 26. Option 4 corresponds to ‘3–4 per week’ for items 1–25 and to ‘5–6’ for item 26. Option 5 corresponds to ‘5+ per week’ for items 1–25 and to ‘7+’ for item 2.

### 3.3. Reliability of the DFS-TR

#### 3.3.1. Internal Consistency

For reliability analysis, the α value for the DFS-TR in adult individuals was 0.777, while the α values for the sub-dimensions ranged between 0.531 and 0.635. The α value for the PFS was 0.863 (Table 3).

#### 3.3.2. Split-Half Reliability

In the study, the items were divided into two single rows using the split-half method. The correlation coefficient between the two halves was 0.681, the Spearman–Brown coefficient was 0.811, and the Guttman coefficient was 0.809 (Table 4).

#### 3.3.3. Test–Retest Reliability

The test–retest analyses revealed that the DFS-TR total score and its sub-dimensions had highly significant (*p* < 0.01) relationships between the two applications, with r values ranging from 0.967 to 0.998 (Table 5).

### 3.4. Known-Groups (Construct) Validity of the DFS-TR

Table 6 presents the average energy and fat, saturated fat, free sugar, and sucrose intakes of participants split into quartiles based on DFS-TR scores. Participants in the top quartile had higher energy and nutrient intakes (*p* < 0.05).

### 3.5. Criterion and Construct Validity

To establish the construct validity of the measurement tool, the relationship between DFS-TR and FFQ and PFS according to gender was evaluated (Table 7). There were positive correlations between the total DFS-TR score and the total energy intake from FFQ (r = 0.445, *p* = 0.000), total fat percentage (r = 0.271, *p* = 0.000), percentage of saturated fat (r = 0.232, *p* = 0.000), sucrose (r = 0.428, *p* = 0.000), and free sugar (r = 0.404, *p* = 0.000). Additionally, significant positive relationships were identified between the total DFS-TR score and the total PFS score (r = 0.298, *p* = 0.000) and the sub-dimension scores.

**Figure 2:**
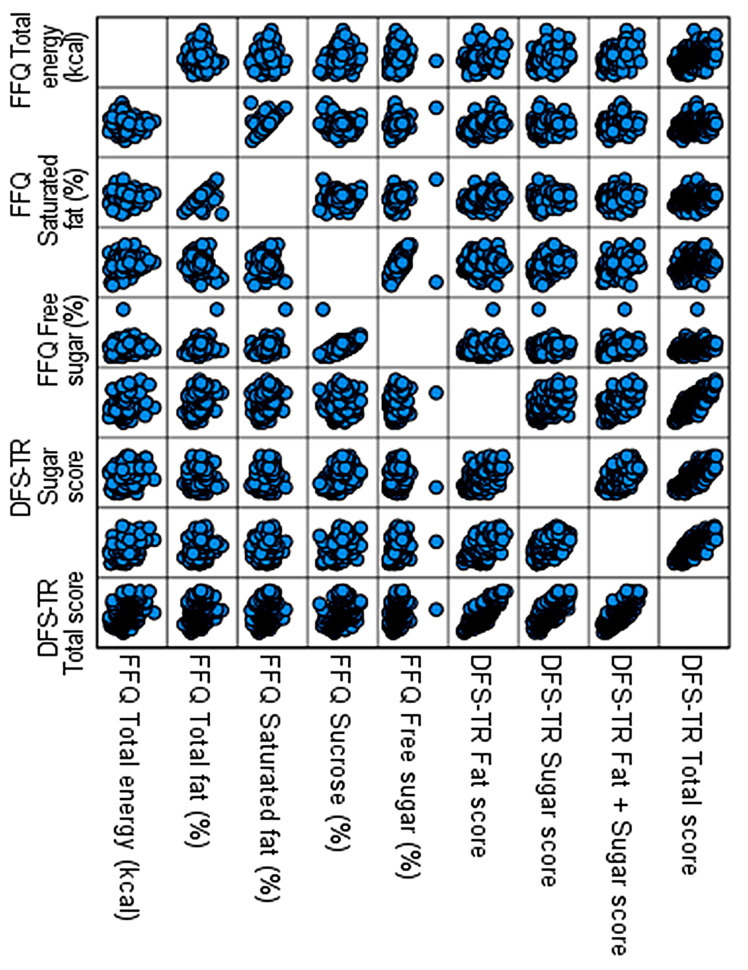
DFS-TR and FFQ related validity.

When evaluated in terms of sub-dimensions, the DFS-TR fat sub-dimension score was associated with the total fat percentage reported in the FFQ (r = 0.359, *p* = 0.000) and the percentage of saturated fat (r = 0.246, *p* = 0.000). There was a significant positive relationship between the DFS-TR sugar sub-dimension score and sucrose (r = 0.468, *p* = 0.000) and free sugar (r = 0.320, *p* = 0.000). Positive significant relationships were also identified between the DFS-TR fat and sugar sub-dimension scores and the total energy intake reported in the FFQ (r = 0.363, *p* = 0.000), total fat percentage (r = 0.231, *p* = 0.000), saturated fat percentage (r = 0.222, *p* = 0.000), sucrose (r = 0.407, *p* = 0.000), and free sugar (r = 0.388, *p* = 0.000) (Table 7, Figure 2).

## 4. Discussion

This study aimed to evaluate the validity and reliability of the Turkish version of the Dietary Fat and Sugar questionnaire (DFS-TR) in a representative adult population. We tested the validity and reliability of the DFS when translated to Turkish, whereby the original questionnaire items were adapted for Turkish specifications. After this adaptation, the questionnaire retained the same number of dimensions and items as in the original English version [17] with three sub-dimensions and 26 items. Similarly to the Polish version of the questionnaire [18], the DFS-TR was culturally adapted to align with local traditions. In the original article, the item regarding pork meat was not supported by those assessing the language validity; therefore, the item was removed because it negatively affected internal consistency [18]. As such, the DFS-TR is viable for other countries of similar culture and dietary patterns and food preferences.

To ensure demographic homogeneity across gender and age groups, the distribution of participants by age was compared with that of the adult population of Turkish women and men [24]. As such, the study was conducted with 314 participants aged 19–64 years (38.78 ± 12.10), of whom 54.5% were female. Generally, participants in the 25–29 years group scored lower on the total DFS-TR score, the fat sub-dimension, and the sugar sub-dimension than participants in other age groups, while individuals with a high school education scored higher. In the German DFS, there was no significant correlation between age and DFS, possibly because of the narrow age range in the sample [4]. In the Polish version, the total DFS score negatively correlated with age (r = −0.15, *p* < 0.05), while the sugar sub-dimension was positively related to age (r = 0.37) and education (r = 0.26; *p* < 0.05) [18]. In our study, no significant differences were observed in total scores, fat, sugar, or the fat + sugar sub-dimensions by gender or marital status (*p* > 0.05). This finding indicates that gender and marital status are not determinants of saturated fat and sugar consumption. As in our study, gender differences did not affect DFS scores in the German DFS, suggesting no significant dietary differences between the Turkish and German cohorts [4]. Conversely, in the Polish DFS, male participants consumed more of the listed products than females. However, no significant difference was found between genders for the consumption of foods rich in both saturated fats and free sugars, referred to as tasty foods (i.e., DFS fat + sugar sub-dimension) [18].

The DFS-TR demonstrated strong reliability and temporal stability. The questionnaire has excellent test–retest reliability (r = 0.967–0.998) and a consistent measurement structure. Additionally, the internal consistency of the total DFS-TR score (α = 0.777) and the sub-dimension α values (α = 0.531–0.635) suggest that the questionnaire is reliable. As such, the questionnaire yielded results similar to those of the original English [17] and other language versions [4,18]. In the Polish adaptation of the questionnaire, test–retest reliability was r = 0.856 and internal consistency was α = 0.797 [18]; in the German version, test–retest reliability was r = 0.801 and internal consistency was α = 0.808 [4].

Additional evidence for internal consistency was provided by split-half reliability analyses. Using this approach, the questionnaire demonstrated a satisfactory level of agreement between the two halves, indicating that the items consistently measure the same underlying construct. This method considers the similarity of the scores obtained from each half of the sample [30]. A correlation coefficient greater than 0.70, that is, close to 1, indicates that the tool is reliable [31]. The correlation coefficient between the two halves of the questionnaire was calculated as 0.681; the Spearman–Brown coefficient was 0.811; and the Guttman coefficient was 0.809, suggesting that the correlation between the two halves is a reliable measure.

Criterion-related validity of the DFS-TR was supported by significant correlations with nutrient intake derived from the FFQ. The original English DFS also showed correlation coefficients ranging from 0.35 to 0.71 when compared with DFS scores, energy derived from saturated fat, and free sugars assessed with the FFQ and a 4-day diet diary [14]. In our study, the total DFS-TR score correlated with total energy intake reported in the FFQ (r = 0.445), as well as with the percentage of total energy from total fat (r = 0.271), saturated fat (r = 0.232), sucrose (r = 0.428), and free sugars (r = 0.404). Additionally, DFS-TR was associated with fat intake, with correlations to the percentage of total fat reported in the FFQ (r = 0.359) and saturated fat (r = 0.246), and to sugars, with correlations to sucrose (r = 0.468) and free sugars (r = 0.320). The presence of significant correlations between the participants’ total DFS-TR scores and sub-dimension scores and their reported nutrient intake from the FFQ indicates that the questionnaire successfully measured the intended items. In the German DFS, similar to this study, there were positive correlations between the total DFS score and reported intake of saturated fat (r = 0.258), free sugars (r = 0.443), and sucrose (r = 0.445) in the FFQ [4]. Likewise, in the Polish version, the DFS showed significant correlations with sugars (r = 0.79), fats (r = 0.75), and fat-sugar (r = 0.59) from the FFQ (*p* < 0.001) [18].

Further support for construct validity was obtained through quartile-based comparisons of dietary intake. Individuals who scored in the lower and upper quartiles of the DFS-TR were compared with respect to their nutrient intake from the FFQ. Those in the upper quartile had higher average intakes of energy, fat, saturated fat, free sugar, and sucrose, and higher total energy and free sugar intake than those in the lower quartile. This result is mirrored in the German DFS, where individuals in the upper quartile of DFS score also had higher total energy and free sugar intake [4].

DFS-TR scores also varied according to sociodemographic, anthropometric, and lifestyle-related characteristics. In our study, the total DFS-TR score of individuals with class 1 obesity was higher than those with normal weight and overweight. Additionally, individuals with obesity scored higher in the sub-dimension of fat, and in the sugar sub-dimension, they scored higher than those with normal weight and overweight. Individuals with obesity scored higher in the combined fat + sugar sub-dimension than those with normal weight, which was an expected observation. This finding suggests that the saturated fat and free sugar intake for those with a BMI > 30 will be well represented, similar to individuals with normal BMI. In the English and Polish versions of the DFS, there was no correlation between the total DFS score and BMI [17,18]. A similar result in the German DFS was reported to be due to individuals with obesity restricting their fat and sugar intake for weight loss purposes or due to underreporting specific to their weight group. Furthermore, the regression coefficients between DFS scores and saturated fat intake decreased with rising BMI [4]. Our findings support the validity and temporal efficiency of the questionnaire in predicting saturated fat and free sugar intake in the general adult population. As the number of main meals consumed increased, the total DFS-TR score and its sub-dimension scores increased. Also, as the number of snacks increased, scores in all sub-dimensions except the fat + sugar sub-dimension and the total DFS-TR also increased. Additionally, individuals who ate outside the home sometimes had higher total, fat sub-dimension, and fat + sugar sub-dimension scores than those who ate at home (*p* < 0.05). Participants who frequently used frying as a cooking method had higher total DFS-TR scores and higher scores on the sugar and fat + sugar sub-dimensions than those who preferred boiling, baking, grilling, or roasting. Those who frequently preferred frying scored particularly high in the fat sub-dimension compared with those who preferred grilling (*p* < 0.05). Individuals who engage in regular physical activity scored significantly lower in the total DFS-TR and in the sugar sub-dimension compared with those who do not exercise or do so occasionally. This study, unlike others, assessed individual differences across sociodemographic, economic, anthropometric, and dietary habits.

In psychometric evaluations, associations between a newly developed or culturally adapted instrument and clinically or psychosocially related measures provide important evidence for structural validity. The statistically significant positive correlations between DFS-TR scores and the PFS shown in the present study support the convergent validity of the questionnaire. At the scale level, the total DFS-TR score showed a moderate association with the total PFS score and its sub-dimensions, and this pattern was consistently observed across the DFS-TR sub-dimensions, including fat, sugar, and fat + sugar. These findings suggest that individuals reporting a higher frequency of consuming fat-rich and sugar-rich foods also exhibit stronger hedonic responses to food-related stimuli. This pattern aligns with the theoretical framework of both instruments: the DFS-TR reflects habitual consumption of energy-dense, palatable foods, while the PFS measures hedonic drive and sensitivity to the rewarding aspects of food. Published research consistently corroborates this relationship. Studies have reported that higher PFS scores are associated with increased intake of sweet, high-fat foods and fast food [32,33] and with greater sugary drink and snack consumption [34,35]. Individuals with higher PFS scores tend to have a stronger drive to eat tasty foods and sometimes lose control of their eating [36]. However, the association between hedonic hunger and actual intake could be influenced by further behavioral and cognitive factors. For example, Naughton et al. (2015) [37] reported that habit strength mediates the relationship between hedonic hunger and sugar consumption, whereas Horwath et al. (2020) [38] found that reduced self-control amplifies the effect of hedonic hunger on the intake of high-fat and high-sugar foods. These findings collectively imply that hedonic hunger is associated with self-regulatory mechanisms and habitual patterns, which together determine whether the motivation to consume palatable foods results in actual intake. In this context, the significant correlations observed between the DFS-TR and PFS in our study further substantiate the biological and psychological plausibility of the DFS-TR. The questionnaire not only captures dietary behavior related to fat and sugar intake, but it also reflects the underlying motivational and hedonic components of eating-related dietary patterns and reward-based eating tendencies observed across diverse populations. When the findings of the study are considered together, they indicate that the DFS-TR could be a psychometrically robust self-reporting tool for predicting saturated fat and free sugar intake among Turkish adults. Furthermore, the use of established, validated, and reliable measurement tools supports the scientific robustness and internal validity of the study outcomes.

The interpretation of the DFS-TR cut-off score requires careful consideration within the Turkish population. In the original validation study, individuals scoring above 60 points—corresponding to the median—were advised to consider reducing their intake of dietary fat and free sugars [17]. In contrast, in the present study, a DFS-TR score above 60 represents the middle and upper quartiles rather than the median. This shift highlights the population-specific nature of cut-off values and supports the adoption of a more conservative interpretation, as previously suggested in other language adaptations [4,27]. Accordingly, although scores above 60 were linked to intake exceeding WHO recommendations in the original study, this threshold should be interpreted in the Turkish context as a pragmatic screening indicator rather than a definitive marker of non-adherence. Interpreting higher DFS-TR scores must also take into account individual differences in energy requirements. Energy needs vary according to anthropometric characteristics, metabolic conditions, and physical activity levels, and individuals with higher energy demands may naturally consume greater quantities of food. As a result, such individuals may report more frequent consumption of foods included in the DFS-TR, leading to higher scores that reflect overall food intake rather than a diet disproportionately rich in saturated fats and free sugars. Therefore, in both clinical and public health settings, DFS-TR scores above this threshold may be most appropriately used to identify individuals who could benefit from further dietary assessment or counseling rather than serving as a diagnostic criterion. These interpretative challenges are further compounded by inherent methodological limitations of dietary assessment tools. Food intake over the previous twelve months was evaluated using both the FFQ and the DFS-TR; however, as the FFQ relies on participants’ long-term memory, it may be particularly susceptible to recall bias, and the exclusive use of self-reported dietary data may increase socially desirable responding. To address these limitations, future studies are encouraged to further examine the validity of the DFS-TR using more objective or repeated dietary assessment methods, such as multiple 24 h dietary recalls or detailed food records. Moreover, the present study did not validate the proposed cut-off against objective dietary intake measures or metabolic or inflammatory outcomes. Therefore, future research incorporating repeated dietary assessments and relevant health biomarkers is warranted to further strengthen the interpretability and practical utility of the DFS-TR as a screening tool in the Turkish population.

Overall, the psychometric properties of the DFS-TR were consistent with those reported in other cultural adaptations of the DFS. The psychometric performance of the DFS-TR is broadly consistent with findings reported in other cultural adaptations of the DFS, including the German and Polish versions [4,18]. Similarly to these studies, the Turkish version demonstrated acceptable internal consistency and strong test–retest reliability, supporting the temporal stability of the instrument. Notably, the test–retest coefficient observed in the present study was higher than those reported in the German and Polish validations, which may reflect differences in sample size, study design, and retest interval rather than substantive differences in measurement quality [4,18]. Across all versions, DFS scores showed significant associations with percentage of energy intake from saturated fat and free sugar assessed by FFQs, supporting convergent validity. While the magnitude of these correlations varied across studies, such differences are likely attributable to methodological variations in dietary assessment tools and population characteristics. Furthermore, the DFS demonstrated theoretically coherent associations with eating-related behavioral constructs across cultures—such as hunger and restraint in the German sample, cognitive restraint and uncontrolled eating in the Polish sample, and food-related responsiveness (i.e., hedonic hunger–related responsiveness to food cues) in the Turkish sample—highlighting the cross-cultural robustness of the underlying construct. Taken together, these findings support the DFS framework as a flexible and culturally adaptable tool for assessing habitual dietary fat and free sugar intake. Considering the methodological limitations reported in previous studies on the DFS-TR, particularly the reliance on small sample sizes and samples with limited representativeness of the general population, the sampling strategy adopted in our study provides a meaningful contribution to strengthening the population-level evidence in the Turkish context. The inclusion of an adequately sized sample stratified according to Turkish Statistical Institute (TÜİK) age and gender distributions enhances the representativeness of the findings [24]. The comprehensive exclusion criteria used in this study were designed to minimize factors that could significantly alter customary dietary fat and free sugar intake, thereby enhancing internal validity. Specifically, individuals with metabolic or systemic diseases, those on fat- or sugar-restricted diets, pregnant or breastfeeding women, elite athletes, and individuals with excessive energy intake were excluded to avoid dietary habits driven by therapeutic, physiological, or performance-related requirements rather than habitual behavior. Similarly, the exclusion of participants with neurological or psychiatric diagnoses, eating disorders, substance dependencies, or formal training in nutrition was performed to reduce response bias and ensure structural clarity in the validation process. While these criteria enhance the psychometric rigor of the DFS-TR, they also indicate that this tool is currently most suitable for the general adult population without specific dietary needs. Therefore, further studies are required in the future to evaluate the performance and applicability of the DFS-TR in clinical populations and other subgroups with different dietary behaviors.

### 4.1. Strengths, Limitations, and Future Directions

This study has several notable strengths. The translation and cultural adaptation of the English DFS followed a rigorous methodological process, including forward–backward translation, linguistic evaluation, and expert panel review, ensuring conceptual and semantic equivalence between the original and Turkish versions. The sample size was sufficient for robust psychometric analyses and was stratified according to national demographic distributions, thereby strengthening representativeness. Data were collected from face-to-face interviews conducted by trained dietitians who were members of the research team, which likely enhanced standardization and improved the quality of dietary assessment. Concurrent validity was assessed using a comprehensive FFQ analyzed with BEBIS software, while the inclusion of the PFS provided additional evidence for convergent validity. Owing to its brevity, cost-effectiveness, ease of administration, and public availability, the DFS-TR appears to be well suited for both clinical and research applications and holds promise as a practical screening tool in large-scale epidemiological and public health contexts.

Nevertheless, several limitations should be acknowledged. Anthropometric data were self-reported, which might have introduced measurement bias in BMI classification. The cross-sectional study design precludes causal inferences and limits conclusions regarding temporal stability. Although face-to-face data collection by dietitians improved consistency, it could also have introduced interviewer bias or socially desirable responses. Participation was voluntary, and despite the use of quota-based sampling, the possibility of self-selection bias remains. In addition, since this study included a sample selected from a single city and according to the age distribution of the country, future studies should include different socioeconomic populations. Although the DFS-TR showed strong psychometric properties, the absence of biochemical or inflammatory measurements restricts the evaluation of its predictive validity for metabolic or inflammatory outcomes. Future research should evaluate the test–retest reliability of the DFS-TR to determine its temporal stability and responsiveness to dietary interventions aimed at reducing fat and free sugar intake. Incorporating biochemical and inflammatory biomarkers—e.g., serum triglycerides, total cholesterol, HbA1c, fasting glucose, C-reactive protein (CRP), interleukin-6 (IL-6), tumor necrosis factor-α (TNF-α), and oxidative stress markers—would strengthen the biological and physiological validity of the questionnaire. Furthermore, investigating the performance of the DFS-TR across diverse subgroups, including individuals with obesity, metabolic syndrome, adolescents, and populations with varying socioeconomic backgrounds, would provide valuable insight into its broader applicability. Regional comparisons within Türkiye could also help assess cultural and dietary pattern differences. Lastly, longitudinal and intervention-based studies could examine whether lower DFS-TR scores correspond to favorable changes in metabolic and inflammatory profiles, supporting the use of the DFS-TR in public health surveillance and preventive nutrition strategies.

### 4.2. Implications for the Practice and Public Health

Beyond its proven reliability and internal consistency, the DFS-TR has various potential applications in clinical nutrition counseling and public health practice when interpreted as a measure of habitual intake rather than short-term dietary behavior. As the scale assesses dietary fat and free sugar consumption over the previous year, it is particularly suitable for identifying long-term dietary habits and sustained exposure relevant to chronic disease risk assessment. Moreover, due to its brevity and focused structure, the DFS-TR enables more rapid identification of saturated fat and free sugar intake compared with lengthy food frequency questionnaires, making it especially suitable for use in busy clinical environments. In clinical settings, the DFS-TR can therefore be employed as a brief and practical screening tool during the initial assessment of nutritional status to identify individuals who frequently consume high levels of dietary fat and free sugars, thereby facilitating the development of personalized counselling strategies. At the population level, the scale may support public health interventions by aiding the identification of high-risk groups and informing the design of targeted nutrition policies. Furthermore, owing to its brevity, low respondent burden, and standardized structure, the DFS-TR can be integrated into national nutrition surveillance systems as a complementary instrument to more detailed dietary assessment methods, such as food frequency questionnaires and dietary records, particularly for monitoring habitual dietary patterns on a large scale.

## 5. Conclusions

Our study confirms that the DFS-TR can be used as a valid and reliable screening tool to assess saturated fat and free sugar intake. The potential use of the DFS-TR in research is important in providing a quick overview of the nutrition of individuals with high or low saturated fat and free sugar consumption. This study supports the validity of the DFS-TR with findings related to internal consistency and test–retest reliability, evaluated across different sociodemographic, economic, anthropometric, and dietary habit variables within the general adult population. Consequently, the questionnaire could be a psychometrically sound self-reporting instrument for predicting saturated fat and free sugar intake among Turkish adults.

## Figures and Tables

**Table 1:** General characteristic of participants.

		n	%
Age (years)	20–24	42	13.4
	25–29	50	15.9
	30–34	41	13.1
	35–39	39	12.4
	40–44	36	11.5
	45–49	34	10.8
	50–54	32	10.2
	55–59	22	7.0
	60–64	18	5.7
Gender	Female	171	54.5
	Male	143	45.5
Education level	Elementary school graduate	11	3.5
	Middle school graduate	43	13.7
	High school graduate	92	29.3
	University graduate	121	38.5
	Postgraduate	47	15.0
Marital status	Married	199	63.4
	Single	115	36.6
Income	Income is less than expenses	26	8.3
	Income equals expenses	208	66.2
	Income is more than expenses	80	25.5
Regular physical activity	Yes	59	18.8
	No	117	37.3
	Sometimes	138	43.9
Physical activities (Multiple markings can be made)	Walking	172	87.3
	Running	11	5.6
	Pilates yoga	21	10.7
	Fitness	22	11.2
	Cardio exercises	20	10.2
	Team sports	20	10.2
BMI (kg/m^2^)	<18.5 (underweight)	13	4.2
	18.5–24.9 (healthy weight)	175	55.7
	25.0–29.9 (overweight)	93	29.6
	30.0–34.9 (class 1 obesity)	29	9.2
	35.0–39.9 (class 2 obesity)	4	1.3

BMI: body mass index.

**Table 2:** Participants’ sociodemographic and anthropometric characteristics and their scores on the DFS-TR according to their dietary habits.

		DFS-TR Total	DFS-TR Fat	DFS-TR Sugar	DFS-TR Fat + Sugar
		Mean ± SD	Mean ± SD	Mean ± SD	Mean ± SD
Age (years)	20–24 (1)	57.10 ± 11.07	28.86 ± 5.48	14.12 ± 3.83	14.12 ± 4.08
	25–29 (2)	52.28 ± 8.45	25.86 ± 4.23	12.44 ± 3.39	13.98 ± 2.97
	30–34 (3)	57.20 ± 8.54	28.27 ± 4.21	13.90 ± 3.58	15.02 ± 3.15
	35–39 (4)	57.69 ± 10.15	29.79 ± 5.14	13.87 ± 4.35	14.03 ± 4.00
	40–44 (5)	60.81 ± 11.06	30.72 ± 5.26	14.92 ± 3.95	15.17 ± 4.67
	45–49 (6)	61.38 ± 9.95	30.09 ± 4.69	15.24 ± 3.89	16.06 ± 4.38
	50–54 (7)	58.06 ± 7.17	28.13 ± 3.38	15.47 ± 3.65	14.47 ± 3.20
	55–59 (8)	57.32 ± 8.19	29.04 ± 4.40	13.28 ± 3.18	15.00 ± 3.20
	60–64 (9)	61.87 ± 13.06	30.40 ± 5.64	16.60 ± 4.98	14.87 ± 4.29
**F**		3.605	4.196	3.231	1.092
***p* value**		0.001 *	0.000 *	0.002 *	0.374
**Post hoc**		2 < 5, 6, 7	2 < 4, 5, 6, 9	2 < 6, 7, 9	
Gender	Female	57.52 ± 10.57	28.49 ± 5.29	14.11 ± 3.92	14.92 ± 3.85
	Male	57.80 ± 9.21	29.13 ± 4.38	14.29 ± 3.95	14.38 ± 3.69
**t**		−0.251	−1.187	−0.410	1.276
***p* value**		0.802	0.236	0.682	0.203
Education level	Elementary school graduate	54.09 ± 3.56	26.55 ± 1.97	14.00 ± 2.14	13.55 ± 2.62
	Middle school graduate	58.23 ± 9.22	28.42 ± 3.69	14.74 ± 4.61	15.07 ± 3.22
	High school graduate	59.15 ± 10.04	29.35 ± 4.92	15.09 ± 3.90	14.72 ± 3.99
	University graduate	57.90 ± 10.98	29.05 ± 5.32	13.95 ± 3.93	14.90 ± 3.76
	Graduate school graduate	54.36 ± 7.87	27.83 ± 5.00	12.62 ± 3.09	13.91 ± 4.10
**F**		4.424	3.794	3.530	0.948
***p* value**		0.003 *	0.007 *	0.008 *	0.437
**Post hoc**		3 > 1, 5	3, 4 > 1	3 > 5	
Marital status	Married	58.45 ± 9.41	29.09 ± 4.73	14.51 ± 4.04	14.85 ± 3.64
	Single	56.27 ± 10.75	28.24 ± 5.15	13.65 ± 3.69	14.37 ± 4.02
**t**		1.874	1.480	1.867	1.073
***p* value**		0.062	0.140	0.063	0.284
Economic level	Income is less than expenses	59.58 ± 8.93	29.96 ± 4.43	14.58 ± 4.54	15.04 ± 4.06
	Income equals expenses	58.10 ± 10.49	28.46 ± 5.04	14.58 ± 4.05	15.06 ± 3.72
	Income is more than expenses	55.85 ± 8.65	29.23 ± 4.61	13.08 ± 3.16	13.55 ± 3.65
**F**		2.476	1.533	5.690	4.871
***p* value**		0.092	0.218	0.005 *	0.008 *
**Post hoc**				2 > 3	2 > 3
Regular physical activity	Yes	54.63 ± 9.99	27.97 ± 5.25	12.47 ± 3.31	14.19 ± 3.89
	No	58.44 ± 9.26	28.82 ± 4.86	14.67 ± 3.93	14.96 ± 3.29
	Sometimes	58.27 ± 10.34	29.09 ± 4.76	14.53 ± 3.99	14.64 ± 4.11
**F**		3.405	1.104	7.289	0.878
***p* value**		0.034 *	0.333	0.001 *	0.418
**Post hoc**		1 < 2		1 < 2, 3	
BMI (kg/m^2^)	<18.5	61.85 ± 9.73	30.54 ± 5.88	16.00 ± 3.11	15.31 ± 3.86
	18.5–24.9	55.46 ± 9.88	27.88 ± 5.03	13.39 ± 3.62	14.18 ± 3.52
	25.0–29.9	58.17 ± 8.48	29.19 ± 3.87	14.11 ± 3.66	14.87 ± 4.16
	30.0–34.9	66.24 ± 9.46	31.31 ± 5.13	18.28 ± 4.02	16.66 ± 3.60
	35.0–39.9	65.50 ± 10.72	34.50 ± 4.04	15.75 ± 5.74	15.25 ± 2.22
**F**		9.755	5.685	11.981	2.980
***p* value**		0.000 *	0.000 *	0.000 *	0.019 *
**Post hoc**		4 > 2, 3	4 > 2	4 > 2, 3	4 > 2
Number of main meals	1	52.95 ± 6.58	25.98 ± 3.44	13.10 ± 3.50	13.88 ± 3.07
	2	56.74 ± 10.08	28.32 ± 4.77	14.02 ± 3.98	14.40 ± 3.75
	3	61.40 ± 9.82	31.10 ± 4.70	14.93 ± 3.94	15.38 ± 3.97
	4 and above	63.67 ± 8.99	30.00 ± 6.34	16.22 ± 2.95	17.44 ± 4.00
**F**		9.269	12.716	3.030	3.569
***p* value**		0.000 *	0.000 *	0.030 *	0.014 *
**Post hoc**		3 > 1, 2; 4 > 1	2, 3 > 1; 3 > 2	4 > 1	4 > 1
Number of snacks per day	0	56.31 ± 9.75	29.46 ± 4.99	12.69 ± 3.22	14.15 ± 3.34
	1	56.46 ± 9.08	28.05 ± 4.48	13.83 ± 3.68	14.58 ± 3.82
	2	58.52 ± 11.10	29.43 ± 5.39	14.51 ± 4.11	14.58 ± 3.80
	3 and above	62.45 ± 9.82	30.69 ± 4.85	15.97 ± 4.49	15.79 ± 3.67
**F**		3.479	3.514	3.378	0.984
***p* value**		0.016 *	0.016 *	0.019 *	0.401
**Post hoc**		3 > 1	3 > 1	3 > 1	
Eating-out habits	Yes	60.01 ± 10.34	29.91 ± 5.18	14.71 ± 3.96	15.39 ± 3.86
	No	62.22 ± 14.58	30.78 ± 4.21	16.78 ± 5.72	14.67 ± 6.69
	Sometimes	53.93 ± 7.68	27.02 ± 3.92	13.27 ± 3.53	13.64 ± 3.14
**F**		16.125	14.820	7.197	8.312
***p* value**		0.000 *	0.000 *	0.001 *	0.000 *
**Post hoc**		1 > 3	1 > 3	1, 2 > 3	1 > 3
Most commonly preferred cooking method	Frying	63.38 ± 10.10	30.64 ± 4.78	16.30 ± 4.23	16.44 ± 3.72
	Boiling	53.88 ± 7.17	27.88 ± 3.79	13.19 ± 3.19	12.81 ± 3.21
	Baking	57.23 ± 10.40	28.82 ± 4.90	13.88 ± 3.64	14.53 ± 4.40
	Steaming	62.67 ± 10.97	30.67 ± 8.96	18.33 ± 3.21	13.67 ± 2.89
	Grilling	53.91 ± 7.29	26.96 ± 3.76	13.13 ± 3.67	13.82 ± 3.21
	Roasting	56.28 ± 9.48	28.47 ± 5.50	13.39 ± 3.56	14.42 ± 2.84
**F**		8.491	4.173	7.377	5.511
***p* value**		0.000 *	0.001 *	0.000 *	0.000 *
**Post hoc**		1 > 2, 3, 5, 6	1 > 5	1 > 2, 3, 5, 6	1 > 2, 3, 5, 6

BMI: body mass index, DFS-TR: Dietary Fat and Free Sugar–Short Questionnaire Turkish version. t: Independent samples *t*-test, F: One-way analysis of variance (ANOVA), * *p* < 0.05.

**Table 3:** Reliability results for measurement tools.

Measurement Tools	Cronbach’s α
DFS-TR	0.777
DFS-TR Saturated fat	0.635
DFS-TR Free sugar	0.532
DFS-TR Saturated fat + free sugar	0.531
PFS	0.863
PFS Food available	0.804
PFS Food present	0.630
PFS Food taste	0.597

DFS-TR: Dietary Fat and Free Sugar–Short Questionnaire Turkish version, PFS: Power oF Food Scale.

**Table 4:** Split-half reliability analysis.

Cronbach’s α	Section 1 = DFS-TR (1), DFS-TR (3), DFS-TR (5), DFS-TR (7), DFS-TR (9), DFS-TR (11), DFS-TR (13), DFS-TR (15), DFS-TR (17), DFS-TR (19), DFS-TR (21), DFS-TR (23), DFS-TR (25)	0.679
	Section 2 = DFS-TR (2), DFS-TR (4), DFS-TR (6), DFS-TR (8), DFS-TR (10), DFS-TR (12), DFS-TR (14), DFS-TR (16), DFS-TR (18), DFS-TR (20), DFS-TR (22), DFS-TR (24), DFS-TR (26)	0.576
Split-half correlation		0.681
Spearman–Brown coefficient		0.811
Guttman split-half coefficient		0.809

DFS-TR: Dietary Fat and Free Sugar–Short Questionnaire Turkish version.

**Table 5:** Test (T1)-retest (T2) results (n = 314).

		Mean	SD	Correlation
DFS-TR Total score	T1	57.65	9.96	0.997 **
	T2	57.58	9.91	
DFS-TR Fat	T1	28.78	4.90	0.994 **
	T2	28.72	4.88	
DFS-TR Sugar	T1	14.19	3.93	0.998 **
	T2	14.17	3.91	
DFS-TR Fat + sugar	T1	14.68	3.78	0.967 **
	T2	12.82	3.49	

DFS-TR: Dietary Fat and Free Sugar–Short Questionnaire Turkish version. Statistical significance was evaluated using Pearson correlation analysis. ** *p* < 0.01.

**Table 6:** Quartiles of DFS-TR scores, ranges, sample size, and average energy intake, along with the percentage of energy derived from saturated fat and free sugars.

		Range Total DFS-TR Score	n	FFQ Total Energy Intake (Kcal)		FFQ Total Fat (%)		FFQ Saturated Fat (%)		FFQ Free Sucrose (%)		FFQ Free Sugar (%)	
				Mean	SD	Mean	SD	Mean	SD	Mean	SD	Mean	SD
DFS-TR	25% (1)	≤51	87	2197.58	419.47	35.97	3.38	17.04	1.65	6.26	1.30	17.26	3.54
	50% (2)	52–56	85	2322.21	394.28	36.70	3.61	17.26	1.82	6.68	1.38	18.27	3.00
	75% (3)	57–63	68	2435.14	548.72	37.31	4.72	17.30	1.85	7.47	1.41	19.55	3.42
	100% (4)	≥64	74	2823.14	601.29	38.60	4.24	18.13	2.10	8.26	1.73	21.51	5.86
**Test value**				19.481 ^F^		6.207 ^F^		5.100 ^F^		26.289 ^F^		47.646 ^KW^	
***p* value**				0.000 *		0.000 *		0.002 *		0.000 *		0.000 *	
**Post hoc**				4 > 1, 2, 3; 3 > 1		4 > 1, 2		4 > 1, 2		4 > 1, 2, 3; 3 > 1, 2		4 > 1, 2, 3	

DFS-TR: Dietary Fat and Free Sugar–Short Questionnaire Turkish version, FFQ: Food Frequency Questionnaire. ^F^ One-way analysis of variance, ^KW^ Kruskal–Wallis H test, * *p* < 0.05.

**Table 7:** Criterion-related validity.

		Total DFS-TR Total Score			DFS-TR Fat Score			DFS-TR Sugar Score			DFS-TR Fat + Sugar Score		
		Female	Male	Total	Female	Male	Total	Female	Male	Total	Female	Male	Total
FFQ total energy intake (kcal)	r_p_	0.470	0.409	0.445	0.395	0.255	0.340	0.384	0.317	0.355	0.359	0.378	0.363
	*p*	0.000 **	0.000 **	0.000 **	0.000 **	0.002 **	0.000 **	0.000 **	0.000 **	0.000 **	0.000 **	0.000 **	0.000 **
FFQ fat (%)	r_p_	0.250	0.302	0.271	0.318	0.424	0.359	0.002	0.030	0.016	0.250	0.218	0.231
	*p*	0.001 **	0.000 **	0.000 **	0.000 **	0.000 **	0.000 **	0.983	0.722	0.776	0.001 **	0.009 **	0.000 **
FFQ saturated fat (%)	r_p_	0.223	0.251	0.232	0.259	0.247	0.246	0.030	0.116	0.069	0.225	0.209	0.222
	*p*	0.003 **	0.002 **	0.000 **	0.001 **	0.003 **	0.000 **	0.692	0.166	0.221	0.003 **	0.012 *	0.000 **
FFQ sucrose (%)	r_p_	0.400	0.477	0.428	0.187	0.187	0.181	0.474	0.469	0.468	0.359	0.466	0.407
	*p*	0.000 **	0.000 **	0.000 **	0.014 *	0.025 *	0.001 **	0.000 **	0.000 **	0.000 **	0.000 **	0.000 **	0.000 **
FFQ free sugar (%)	r_s_	0.412	0.405	0.404	0.302	0.128	0.221	0.348	0.307	0.320	0.327	0.457	0.388
	*p*	0.000 **	0.000 **	0.000 **	0.001 **	0.014 *	0.000 **	0.000 **	0.047 *	0.000 **	0.000 **	0.000 **	0.000 **
PFS total score	r_p_	0.351	0.233	0.298	0.400	0.121	0.282	0.224	0.155	0.192	0.188	0.271	0.222
	*p*	0.000 **	0.005 **	0.000 **	0.000 **	0.150	0.000 **	0.003 **	0.064	0.001 **	0.014 *	0.001 **	0.000 **
PFS food available score	r_p_	0.369	0.263	0.325	0.433	0.178	0.330	0.198	0.190	0.195	0.217	0.241	0.225
	*p*	0.000 **	0.002 **	0.000 **	0.000 **	0.033 *	0.000 **	0.010 **	0.023 *	0.001 **	0.004 **	0.004 **	0.000 **
PFS food present score	r_p_	0.287	0.312	0.296	0.291	0.214	0.257	0.223	0.193	0.209	0.162	0.320	0.231
	*p*	0.000 **	0.000 **	0.000 **	0.000 **	0.010 *	0.000 **	0.003 **	0.021 *	0.000 **	0.034 *	0.000 **	0.000 **
PFS food taste score	r_p_	0.220	0.033	0.142	0.266	−0.079	0.127	0.148	0.020	0.091	0.087	0.153	0.114
	*p*	0.004 **	0.699	0.012 *	0.000 **	0.351	0.024 *	0.053	0.809	0.108	0.255	0.068	0.043 *

DFS-TR: Dietary Fat and Free Sugar–Short Questionnaire Turkish version, FFQ: Food Frequency Questionnaire, PFS: Power of Food Scale. * *p* < 0,05, ** *p* < 0,01. r_s_: Spearman correlation analysis; r_p_: Pearson’s correlation analysis.

## Data Availability

Dataset available upon request from the authors due to privacy.

## Supplementary Materials

The following supporting information can be downloaded at: https://www.mdpi.com/article/10.3390/nu18030421/s1, Table S1: Diyet Yağı ve Serbest Şekeri–Kısa Anketi Türkçe Versiyonu (DYSŞ-TR); Table S2: English Translation of the Turkish Adaptation of the Dietary Fat and Free Sugar–Short Questionnaire (DFS-TR).
