# Supplementary material for: Psychometric Properties of the Turkish Version of the Dietary Fat and Free Sugar-Short Questionnaire (DFS-TR) in Adults: A Validity and Reliability Study
Source: Nutrients. 2026 Jan 27;18(3):421. doi: 10.3390/nu18030421 (PMC12899756; doi:10.3390/nu18030421)
Supplement: Supplementary file 1 [file nutrients-18-00421-s001.zip › nutrients-4065378 - Supplementary.pdf]

nutrients

MDPI

Supplementary Materials

Table S1. Diyet Yağı ve Serbest Şekeri – Kısa Anketi Türkçe Versiyonu (DYSŞ-TR)[1]

Geçen yıl yediğiniz yiyecekleri düşünün. Kahvaltıyı, öğle yemeğini, akşam yemeğini ve dışarıda yediğiniz yemekleri hatırlayın. Lütfen aşağıdaki yiyecek ve içecekleri ne sıklıkta tükettiğinizi en iyi tanımlayan seçeneği işaretleyiniz.

		Ayda 1 kez ve daha az	Ayda 2-3 kez	Haftada 1-2 kez	Haftada 3-4 kez	Haftada 5 kez ve daha fazla
1	Kıyma, dana eti veya kuzu eti (örneğin hamburger, pide, lahmacun, döner, kebap veya ev yemekleri içerisinde bulunan et)					
2	Sandviç içinde veya biftek, kaburga, rosto şeklinde dana eti					
3	Fırında tavuk, kızarmış tavuk veya tavuk burger gibi tavuk içeren tüm yemekler					
4	Sucuk, salam, sosis					
5	Pastırma, füme etler					
6	Salata sosları (az yağlı olanlar hariç)					
7	Margarin, tereyağı, sıvı yağlar veya yemek pişirmede kullanılan diğer yağlar					
8	Yumurta					
9	Pizza					
10	Peynir, sürülebilir peynir (az yağlı olanlar hariç)					
11	Patates kızartması					
12	Mısır cipsi, patates cipsi, yağlı patlamış mısır					
13	Çikolata veya krema dolgulu tatlılar, pastalar, şerbetli tatlılar, hamur işleri					
14	Kekler, kurabiyeler					
15	Dondurma [sorbe (şekerli buzlu meyve) veya az yağlı olanlar hariç]					
16	Çikolata					
17	Şekerlemeler (örneğin lokum, akide şekeri gibi)					
18	Sürülebilir fındık veya fıstık ezmesi, reçel, marmelat, bal					
19	Pankek, waffle					
20	Sporcu içecekleri (örneğin Powerade, Gatorade), enerji içecekleri (örneğin Red Bull)					
21	Alkolsüz içecekler (diyet içecekler hariç)					
22	Süt (sadece tam yağlı)<br>Tek başına veya cappuccino, milkshake, sıcak çikolata gibi içecekler içindeki süt					
23	Diğer tatlandırılmış içecekler (örneğin şeker ilaveli meyve suyu, alkolsüz konsantre şurup, şekerli çaylar)					
24	Beyaz ekmek					
25	Geçen yıl paket servisi hizmeti veren restoranlardan, Meksika, Çin, Tayland, İtalyan (pizza veya makarna) restoranlarından veya fast food restoranlarından kaç kez yemek yediniz?					
		Hiç	1-2	3-4	5-6	7+
26	Geçen hafta içeceklerinize veya besininize/yemeğinize kaç çay kaşığı şeker eklediniz?					

[1] Permission to use this scale must be obtained from the author.

Table S2. English Translation of the Turkish Adaptation of the Dietary Fat and Free Sugar–Short Questionnaire (DFS-TR)[2]

Think about the foods you have consumed over the past year. Include all meals: breakfast, brunch, lunch, dinner, afternoon tea, and supper. For each of the following foods and drinks, evaluate how often you consumed it, indicating only one answer.

		Once a month or less	2–3 per month	1–2 per week	3–4 per week	5+ per week
1	Minced meat, beef or lamb (for example meat in foods such as hamburger, "pide", "lahmacun", doner, kebab or in home-cooked dishes)					
2	Beef in sandwiches or served as steak, ribs or roast					
3	All dishes containing chicken, such as baked chicken, fried chicken or chicken burger					
4	"Sucuk", salami, sausages					
5	"Pastırma", smoked meats					
6	Salad dressings (excluding low-fat varieties)					
7	Margarine, butter, vegetable oils or other fats used in cooking					
8	Eggs					
9	Pizza					
10	Cheese, spreadable cheese (excluding low-fat varieties)					
11	French fries					
12	Corn chips, potato chips, oil-popped popcorn					
13	Chocolate- or cream-filled desserts, cakes, syrup-based/traditional sherbet desserts, and pastries					
14	Cakes, biscuits and cookies					
15	Ice cream [excluding sorbet (sugary iced fruit) or low-fat varieties]					
16	Chocolate					
17	Confectioneries (for example Turkish delight, traditional hard candy such as "akide şekeri")					
18	Spreadable hazelnut or peanut/pistachio butter, jam, marmalade, honey					
19	Pancakes, waffles					
20	Sports drinks (for example Powerade, Gatorade), energy drinks (for example Red Bull)					
21	Non-alcoholic beverages (excluding diet drinks)					
22	Milk (full fat only)<br>Milk consumed on its own or as part of beverages such as cappuccino, milkshake or hot chocolate					
23	Other sweetened beverages (for example sugar-sweetened fruit juice, non-alcoholic concentrated syrup, sweetened teas)					
24	White bread					
25	In the past year, how often did you eat meals from restaurants offering delivery services, including Mexican, Chinese, Thai, Italian (pizza or pasta), or fast-food restaurants?					
		None	1-2	3-4	5-6	7 +
26	In the past week, how many teaspoons of sugar did you add to your beverages or foods/meals?					

[2] This is only a translation of Turkish DFS content. It cannot be used in research in this English version. For the validated English version, please use Francis and Stevenson (2013).
